# Chemotherapy-Induced Tunneling Nanotubes Mediate Intercellular Drug Efflux in Pancreatic Cancer

**DOI:** 10.1038/s41598-018-27649-x

**Published:** 2018-06-21

**Authors:** Snider Desir, Patrick O’Hare, Rachel Isaksson Vogel, William Sperduto, Akshat Sarkari, Elizabeth L. Dickson, Phillip Wong, Andrew C. Nelson, Yuman Fong, Clifford J. Steer, Subbaya Subramanian, Emil Lou

**Affiliations:** 1 Department of Medicine, Division of Hematology, Oncology and Transplantation, University of Minnesota, Minneapolis, MN 55455 USA; 2 Department of Integrative Biology and Physiology, University of Minnesota, Minneapolis, MN 55455 USA; 3 Department of Obstetrics and Gynecology, Division of Gynecologic Oncology, University of Minnesota, Minneapolis, MN 55455 USA; 4 Department of Medicine, Division of Gastroenterology, Hepatology and Nutrition, University of Minnesota, Minneapolis, MN 55455 USA; 5 Department of Laboratory Medicine & Pathology, University of Minnesota, Minneapolis, MN 55455 USA; 6 Department of Surgery, City of Hope Medical Center, Duarte, CA 91010 USA; 7 Department of Surgery, University of Minnesota, Minneapolis, MN 55455 USA

## Abstract

Intercellular communication plays a critical role in the ever-evolving landscape of invasive cancers. Recent studies have elucidated the potential role of tunneling nanotubes (TNTs) in this function. TNTs are long, filamentous, actin-based cell protrusions that mediate direct cell-to-cell communication between malignant cells. In this study, we investigated the formation of TNTs in response to variable concentrations of the chemotherapeutic drug doxorubicin, which is used extensively in the treatment of cancer patients. Doxorubicin stimulated an increased formation of TNTs in pancreatic cancer cells, and this occurred in a dose-dependent fashion. Furthermore, TNTs facilitated the intercellular redistribution of this drug between connected cells in both pancreatic and ovarian cancer systems *in vitro*. To provide supportive evidence for the relevance of TNTs in pancreatic cancer *in vivo*, we performed multiphoton fluorescence microscopy and imaged TNTs in tumor specimens resected from three human patients with pancreatic adenocarcinoma, and one with neuroendocrine carcinoma. In sum, TNT formation was upregulated in aggressive forms of pancreatic carcinoma, was further stimulated after chemotherapy exposure, and acted as a novel method for drug efflux. These findings implicate TNTs as a potential novel mechanism of drug resistance in chemorefractory forms of cancer.

## Introduction

Cancer cells are, in part, characterized by their capacity for invasion. They reside in a context of heterogeneous and stroma-rich tumor microenvironments. The concept of tumor heterogeneity to drug resistance in cancer treatment is well-established^1^. This property includes heterogeneity within the same cancer type between patients, inter-tumoral heterogeneity between different tumors (primary or metastatic) within the same patient, and intra-tumoral heterogeneity within any given single tumor. Tumor-stromal proportion may vary widely and correlates with patient prognosis^2–4^. The relationship and interaction between malignant and stromal cells is dynamic and in constant flux, as cancer cells react and respond to metabolic and physiologic stressors from each other and from the surrounding environment.

Intercellular communication has gained increasing attention as a critical factor to induce heterogeneity in the tumor microenvironments. The effects of direct cell-to-cell transfer of signals (via horizontal transfer) have long been understood to occur via soluble signals such as chemokines and cytokines. More recently, they have been investigated via cellular channels or carriers such as gap junctions and extracellular vesicles (EVs), including exosomes and microvesicles. These physical modes of cellular communication are responsible for transmission of key signals of cellular proliferation and growth that permit tumor progression. Also, their expression appears to be modulated in response to external signals, including exposure to drugs administered with therapeutic intent.

A relatively new form of intercellular communication known as tunneling nanotubes (TNTs) represents an addition to the cadre of physical mechanisms of cellular signaling^5,6^. These structures are long, thin (50–1000 nm in width) F-actin-based cellular protrusions, allowing cells connected by TNTs to perform efficient and direct cell-to-cell transfer of cytoplasmic signals, including mitochondria, microRNAs, and other cellular components^7–30^. TNTs are upregulated in invasive forms of cancer as compared to stromal or non-malignant cells^19,31^ and are induced *in vitro* after exposure to metabolic or physiologic forms of stress, including serum-deprivation, hypoxia, hyperglycemia, and hydrogen peroxide^19,32–34^. We hypothesized that TNTs may be further upregulated after exposure to chemotherapeutic drugs and may represent a unique form of cellular stress response that permits cells to redistribute drugs, thereby reducing the overall kill rate of cancer cells. Here, we present data demonstrating variable formation of TNTs after exposure to the anthracycline chemotherapeutic agent, doxorubicin, in pancreatic and ovarian cancer models and examine the effects of intercellular redistribution of doxorubicin via TNTs. Our findings show that TNTs have the ability to effectively redistribute a chemotherapeutic drug. Such redistribution via TNTs could be a potential mechanism for emergence of chemotherapeutic drug resistance in cancer.

## Results

### TNTs can be visualized in intact malignant pancreatic tumors resected from human patients: supportive evidence that TNTs are an *in vivo* phenomenon

There is significant heterogeneity in tumor-stroma proportions between patients, even within the same type of cancer. This biologic characteristic poses a challenge to achieving uniform efficacy of targeted therapeutics in many solid tumor types. This is especially true in pancreatic carcinomas, in which the stromal matrix is particularly dense as a result of desmoplastic reaction that takes places throughout the process of tumor formation^35,36^. As a direct result, malignant cells are often separated by distance, and thus they are not located in immediate proximity; this characteristic makes reliance on gap junction-mediated intercellular communication prohibitive. Furthermore, the vast majority (90–95%) of pancreatic adenocarcinomas harbor mutant forms of the KRAS oncogene^37–39^. Cells harboring mutant KRAS do not form connexin-lined gap junctions^40–43^. Exosomes have been implicated as one form of long-distance cellular signaling in pancreatic cancer^44^. In this context, there is also a clear niche for long-range cell communication that can be further explained by formation of TNTs.

**Figure 1:**
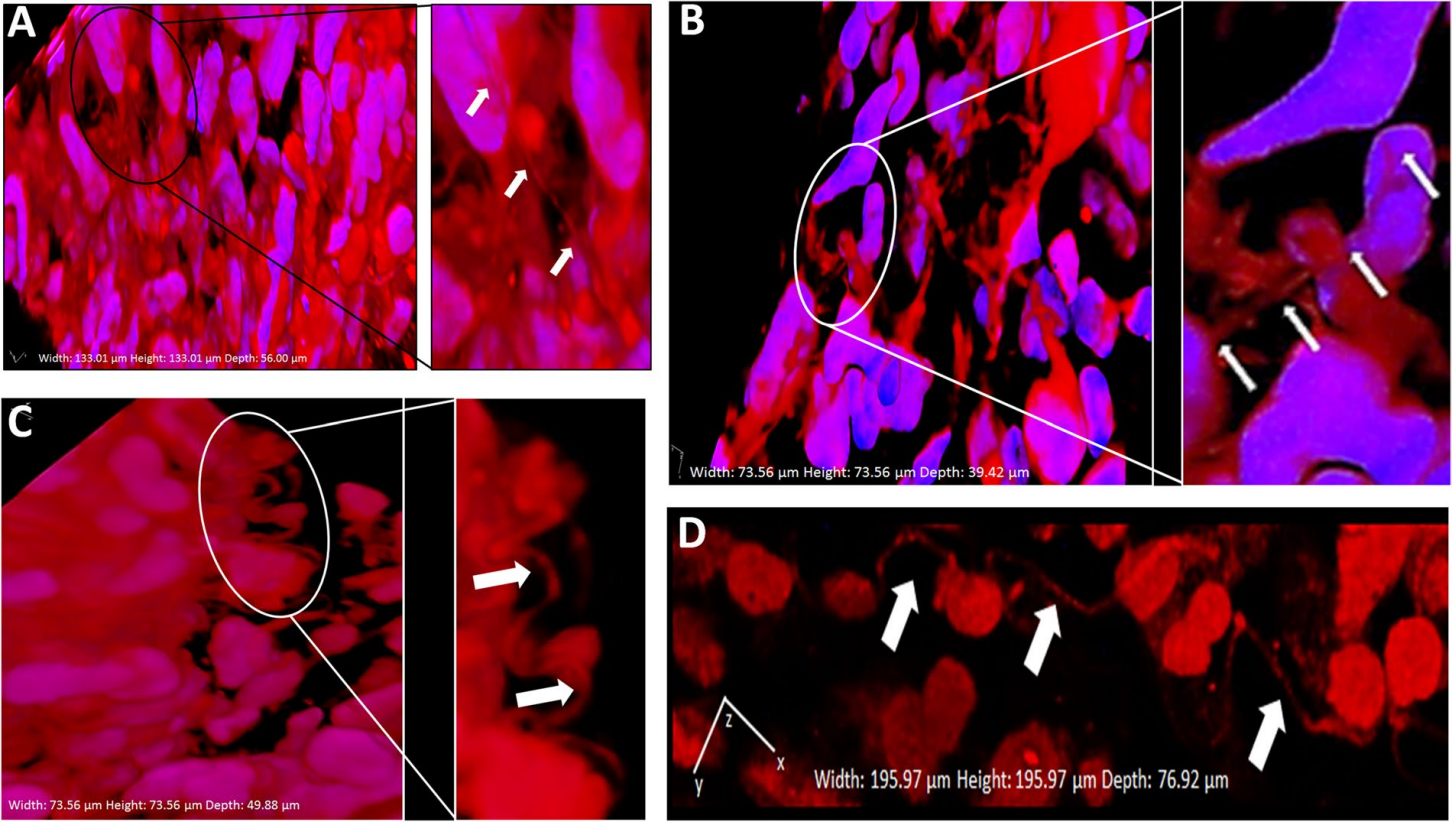
TNTs are identified in resected human pancreatic carcinomas. Tunneling nanotube-like structures, likely TNTs, were visualized connecting cells in tissue samples resected from pancreatic ductal adenocarcinoma patients. The tumors shown in this figure were stained with fluorescing MitoTracker Orange dye and imaged using confocal microscopy with z-stacking of images under 40x oil objective lens. The average z-stack distance (z-step) was 0.42 µm/slice; 110 slices were imaged, for a total z-range of 46.20 µm. 3-dimensional reconstruction was done using NIS elements AR (version 4.00.07) software analysis (Nikon Instruments, Inc, Melville, NY) and included volumetric XYZ cross-sectional planes as shown. (**A**) Highly dense desmoplastic stroma is seen at low magnification; inset shows a TNT (delineated by arrows) at higher magnification. Panels B,C,D Similar examination of more highly curved TNTs/TNT-like extensions connecting cells within the dense matrix of the intact tumor microenvironment.

**Figure 2:**
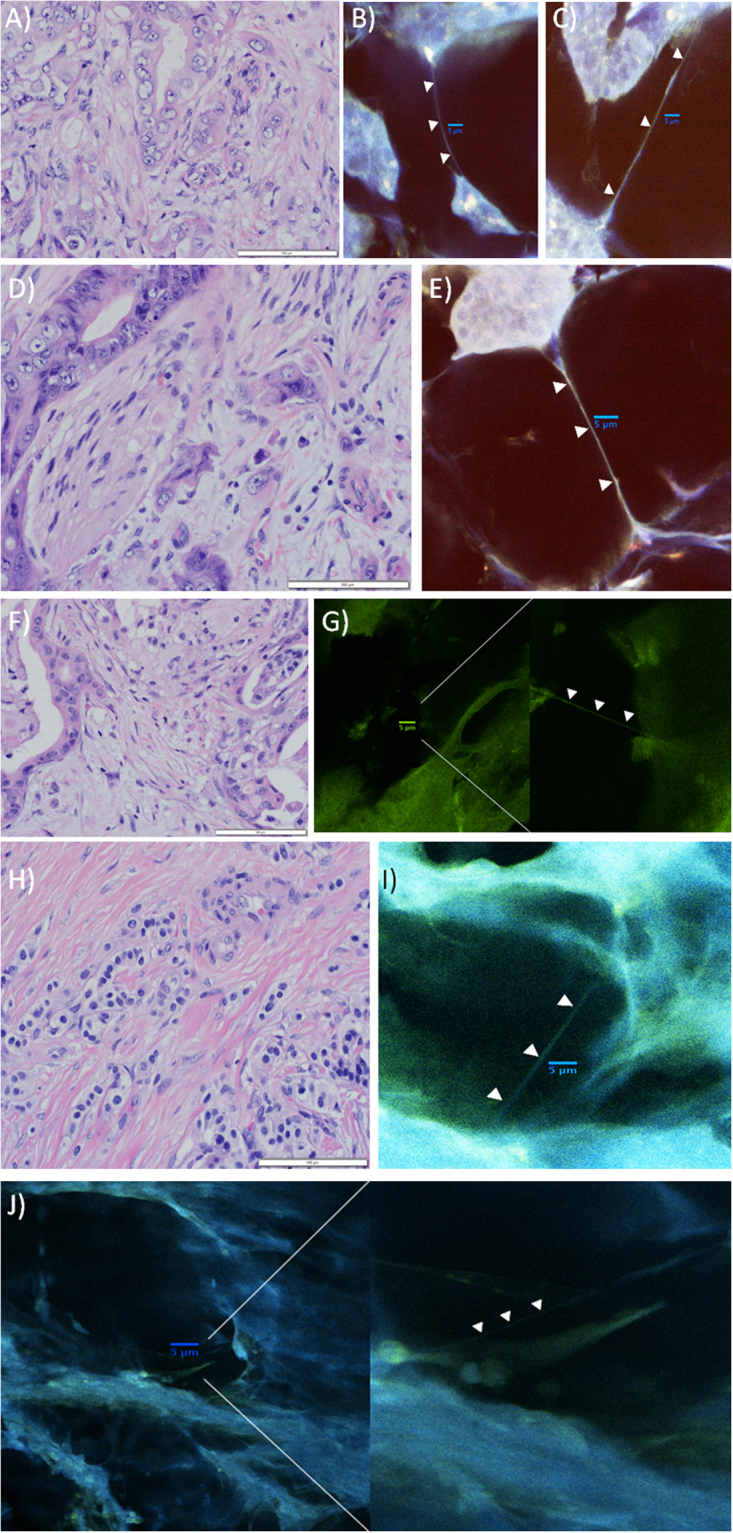
Examples of 3-dimensional imaging revealing TNTs in pancreatic cancer tissue from three additional patients (two with pancreatic adenocarcinoma, one with pancreatic neuroendocrine tumor), using modified techniques intended to improve identification of TNTs amidst the dense stroma. All of these confocal images were acquired using Nikon A1R Multiphoton confocal microscope at 25x with a water immersion objective lens. Images from the tumor from the second patient are shown in panels A–E; F & G are from the third patient; (**H–J**) are from the fourth patient. Images of the tumor from the first patient cited in the text are shown in Fig. 1. (**A**) Hematoxylin and Eosin (H&E) stain of resected pancreatic adenocarcinoma from a 82 year-old male patient, demonstrating islands of malignant cells separated by cellular stroma with moderate collagen deposition, characteristic of this form of cancer (40x objective). The tumor was a 1.2 cm hypoechoic mass in the pancreatic head. The patient received initial treatment with neoadjuvant (preoperative) gemcitabine chemotherapy, followed by pancreaticoduodenectomy (Whipple procedure). Prior to starting adjuvant (post-operative) chemotherapy treatment, he was found to have new liver lesions that were confirmed by biopsy to be indicative of rapid recurrence of a particularly aggressive tumor. Scale bar = 100 μm. (**B**) Confocal imaging of the same tumor revealed TNTs connecting individual or groups of malignant cells. The tumor was sectioned into 100 μm thick slices using a microtome; these sections were stained using 0.5 μm MitoTracker Orange and 0.12 μm Hoechst nuclear dye (no Triton-X was used in preparation of this section). Scale bar = 5 μm. (**C**) A TNT connecting two islands of pancreatic carcinoma cells can be visualized using confocal imaging. This section was prepared using Triton-X with the same stains as used for the image in B. Scale bar = 5 μm. (**D**) Another H&E stained image of the same resected tumor (40 × objective), depicting perineural invasion by two malignant glands. Additional islands of malignant cells are separated by a large amount of dense cellular and collagenous stroma. Scale bar = 100 μm. (**E**) Confocal image depicting a long TNT connecting an island of malignant pancreatic cells to another group of cells at long-range. In contrast to panel D, this image exemplifies the fact that TNTs are not visualized in two-dimensional imaging using standard histopathology preparation techniques, but they can be visualized following a more deliberate and meticulous preparation protocol and approach to confocal imaging as described here. Scale bar = 5 μm. (**F**) H&E stained image of a second tumor, a moderately differentiated pancreatic head adenocarcinoma resected from a 75 year-old male patient. The tumor extended beyond the pancreas and invaded the duodenal wall and peripancreatic adipose tissue (40 × objective). The morphology is similar, showing malignant glands scattered throughout copious desmoplastic stroma. Scale bar = 100 μm. (**G**) Confocal imaging of the tumor from panel F. The tumor was sectioned into 300 μm slices, stained with 1.98 μm Phalloidin and 0.12 μm Hoechst dye, and imaged using 40 z-stacks (z-stack step size 0.3 μm). The panel on the right is a close-up of the image shown in the left panel; the TNT was initially difficult to visualize amidst a densely desmoplastic stromal microenvironment. Scale bar = 5 μm. (**H**) H&E stained image from a 66 year-old woman with a low-grade neuroendocrine tumor of the pancreas. The tumor invaded the ampulla and the duodenal wall in the setting of chronic pancreatitis, with evidence of lymphovascular and perineural invasion on histopathologic examination. Small alveoli of neuroendocrine carcinoma cells are showing streaming through a densely collagenous stroma. Scale bar = 100 μm. (**I**) Confocal imaging of the pancreatic neuroendocrine tumor. Tumor specimen was sectioned into 300 μm slices, stained with 2.97 μm Phalloidin and 0.12 μm Hoechst dye. Scale bar = 5 μm. (**J**) Additional image of the neuroendocrine tumor shown in panels H and I, shown in a higher-magnification view in the panel on the right. Scale bar = 5 μm.

As a demonstration of the potential *in vivo* relevance of TNT investigations to human pancreatic adenocarcinomas, we obtained primary tumor specimens from four patients with resected malignant pancreatic tumors at the time of Whipple surgery (pancreaticoduodenectomy) (Figs 1 and 2). The first specimen, as shown in Fig. 1, was resected from a 75-year-old patient with a pathologically staged T3 tumor (invasive pancreatic carcinoma extending beyond the pancreas, with invasion of duodenal submucosa and peripancreatic adipose tissue), exhibiting poor prognostic features including lymphovascular and perineural invasion and positive lymph nodes in the setting of chronic pancreatitis. This patient received neoadjuvant chemotherapy treatment (gemcitabine) prior to surgical resection.

Imaging this sample revealed the dense stromatous microenvironment that is characteristic of the desmoplastic reaction seen in pancreatic carcinomas. The thickness of this environment presented more challenges in visualizing TNTs/TNT-like structures in this context, compared to other invasive solid tumor malignancies. For this reason, we further adapted our protocol by using finely sectioned thin tumor slices (100 and 300 µm in thickness), and were able to visualize more distinct TNTs connecting cells in tumors resected from two additional patients with localized pancreatic adenocarcinoma, as well as a fourth patient with pancreatic neuroendocrine tumor in the setting of chronic pancreatitis (Fig. 2). The median TNT length was determined from serial images to be 30–40 µm in length in these tumors (Supplementary Fig. 1).

### Pancreatic adenocarcinoma cells form more TNTs than pancreatic ductal epithelium

**Figure 3:**
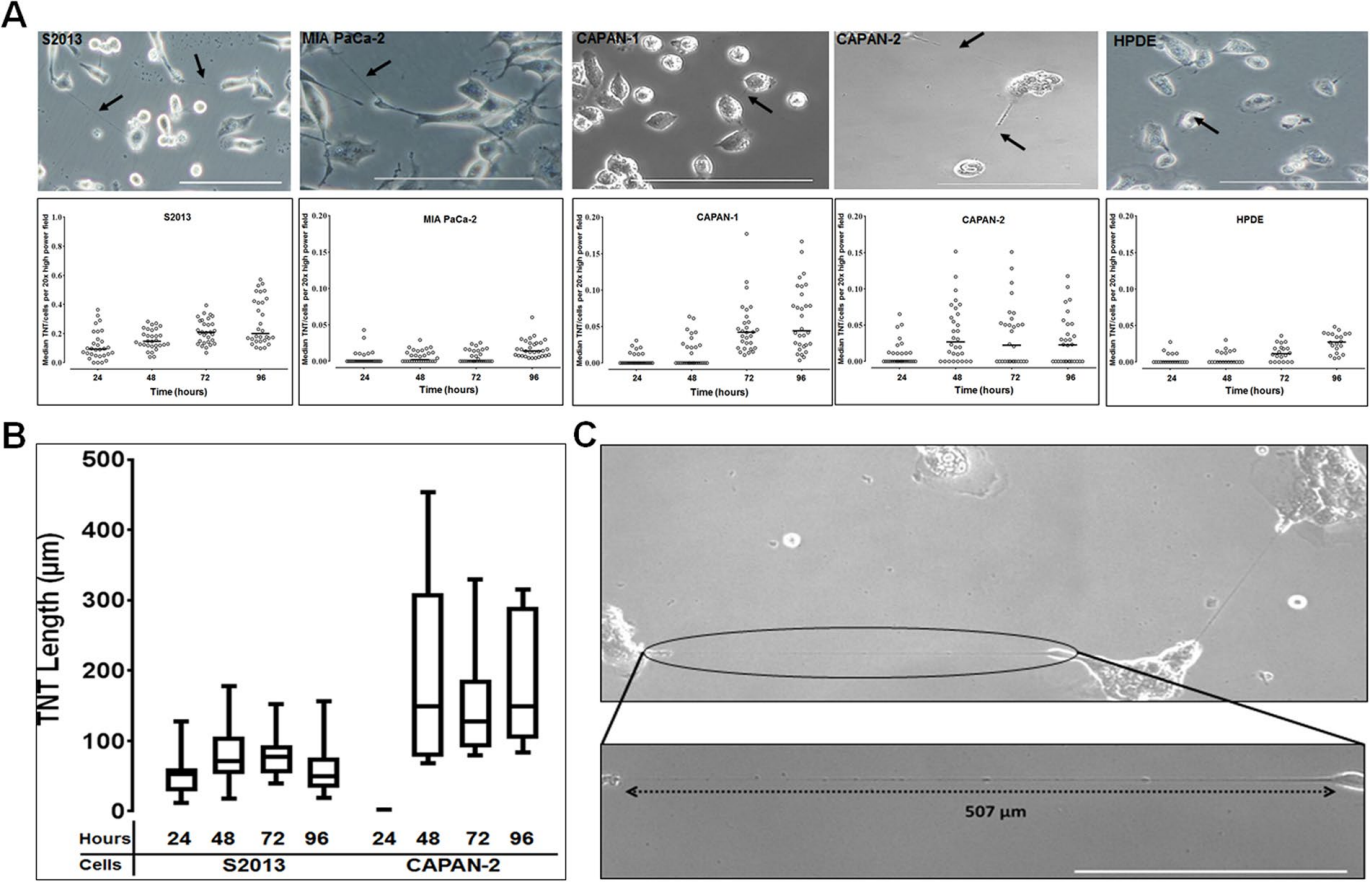
TNT formation amongst pancreatic cancer cells. (**A**) Top row: Representative images of TNTs connecting pancreatic adenocarcinoma S2013, CAPAN-1, CAPAN-2, MIA PaCa-2, and human pancreatic ductal epithelial (HPDE) cells. Images were taken using an Olympus IX70 inverted microscope. Scale bar = 200 μm. Bottom row: The median number of TNTs/cell (TNT index) over time for the S2013, CAPAN-1, CAPAN-2, MIA PaCa-2, and HPDE cell lines. Individual data points are depicted as shown, with solid lines representing median values. (**B**) Box plots depicting the length of TNTs formed in S2013 and CAPAN-2 cells every 24 hours, up to 96 hours. The box indicates the 25^th^ to 75^th^ percentiles, the solid line represents the median, and the whiskers represent the minimum and maximum values. (**C**) High-magnification view of particularly long TNTs (TNT shown in inset exceeds 500 µm in length) connecting CAPAN-2 pancreatic carcinoma cells (magnification = 1.5 times 20x objective).

We have previously quantitatively examined the formation of TNTs in a variety of cancer cell types *in vitro*, including malignant pleural mesothelioma and ovarian cancer^31,32^. To determine the TNT index (i.e., average number of TNTs per cell), we cultured four commonly used pancreatic adenocarcinoma cell lines (MIA PaCa-2, S2013, CAPAN-1, and CAPAN-2) in medium previously reported to stimulate formation of TNTs (Fig. 3A)^19,31^. Of these cell lines, 2 are derived from primary pancreatic tumors (MIA PaCa-2, CAPAN-2) and the other 2 are derived from metastatic lesions (S2013, CAPAN-1). For comparison to non-malignant cells, we also evaluated HPDE, a cell line derived from pancreatic ductal epithelium. Formation of TNTs was negligible in HPDE cells.

TNTs and cells were counted at 24, 48, 72, and 96 hours; data were summarized as the median number of TNTs/cell at these time intervals since TNT formation is not normally distributed (Fig. 3A; cell proliferation curve in Supplementary Fig. 2). Further viewing the data graphed using the mean values of TNTs/cell also demonstrated the differences in TNT formation between malignant cell lines S2013, CAPAN-1, and CAPAN-2 as compared to HPDE cells; formation of TNTs in the MIA PaCa-2 cell line was negligible (Supplementary Fig. 3). We detected heterogeneity in the rates of TNT formation across malignant cell lines, consistent with prior reports from our group of such heterogeneity in cell lines from similarly invasive cancers such as malignant mesothelioma^31^. For HPDE cells, the TNT indices were significantly greater at 72 hours (p = 0.02) and 96 hours (p < 0.0001) than at 24 hours, but not significantly different between 48 and 24 hours (p = 1.00). In a separate experiment, we compared HPDE cells grown in standard culture medium (Keratinocyte-SFM supplemented with epidermal growth factor (EGF) and bovine pituitary extract), compared with HPDE cells grown in TNT-inducing medium, and found no difference in TNT formation between these two conditions (Supplementary Fig. 4).

There were no statistically significant differences in TNTs/cell over time between the metastatic and primary-derived malignant cell lines. For comparison of TNT formation over time, we specifically analyzed the differences between S2013 and MIA PaCa-2 over time as follows: for S2013, TNT indices were significantly greater at 48 hours (p = 0.02), 72 hours (p < 0.0001), and 96 hours (p < 0.0001) than at 24 hours. For MIA PaCa-2, TNT indices were significantly greater at 48 hours (p < 0.0001), 72 hours (p < 0.0001), and 96 hours (p < 0.0001) than at 24 hours.

To assess the distance across which the cells were forming TNTs, we measured TNT lengths over time. Of the cell lines used in this study, we were able to identify enough TNTs in randomly acquired images for S2013 and CAPAN-2 to measure TNT lengths every 24 hours over a 96-hour period. Data are presented as box plots to illustrate the range of TNT lengths during this duration (Fig. 3B). We found the TNTs were longest for both S2013 and CAPAN-2 at 48-hours. Although the lengths varied, the difference in the median TNT length for the individual cell lines was not significant over the entire 96-hour period. However, consistent with heterogeneity shown in other cancer types in our previously reported evaluation of TNT lengths^31^, the CAPAN-2 cell line formed longer TNTs than S2013 by 48-hours in culture. An example of a particularly long TNT, exceeding 500 µm and nearly 4-fold the length of the diameters of the cells it is connecting, is shown as a visual example of the distance that some TNTs can traverse (Fig. 3C).

### TNTs form in a dose-dependent fashion after exposure to doxorubicin

**Figure 4:**
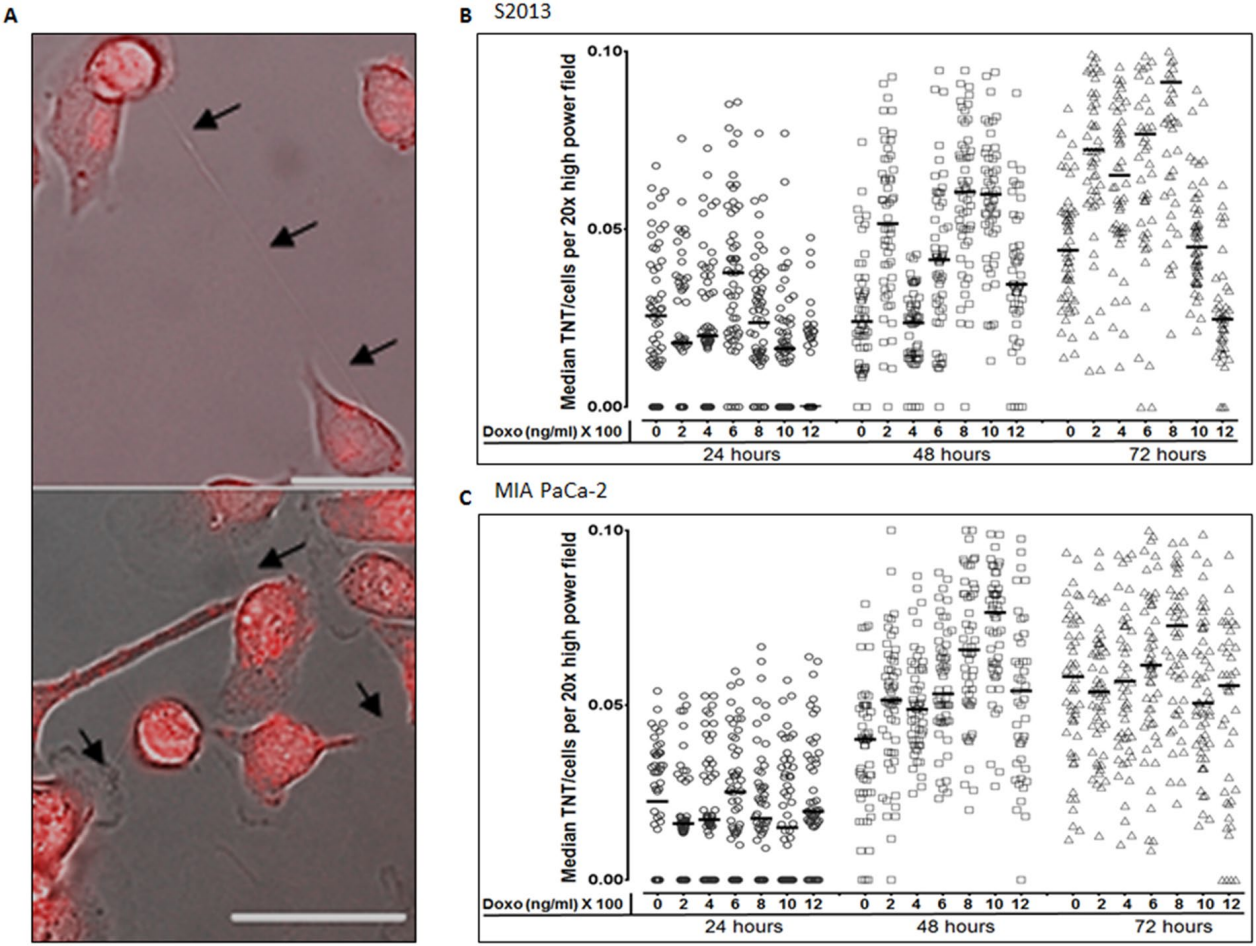
Variable dose-dependent response of TNTs forming after exposure to the chemotherapeutic drug doxorubicin. (**A**) Representative images of TNTs transmitting autofluorescing (red) doxorubicin between S2013 cells. Images were taken using a Zeiss Axio widefield microscope. Scale bars = 50 μm. (**B**,**C**) Scatter-plot graph depicting the median number of TNTs/cell (TNT index) over time, comparing results for the S2013 (Panel B) and MIA PaCa-2 (Panel C) cell lines after exposure to six concentrations of doxorubicin. This experiment was done in triplicate, with a sample size of 18 fields of view per dose/per time point.

Having confirmed a higher rate of TNT formation in pancreatic carcinoma cell lines as compared with ductal epithelial cells, we next examined the effects of chemotherapeutic drug exposure on formation of TNTs between these cells. We focused on examining MIA PaCa-2 and S2013 as we had found statistically significant differences in TNT formation at baseline. We cultured these malignant cells in the presence of six different concentrations of doxorubicin, an anthracycline chemotherapeutic drug that has been used extensively for *in vitro* studies of pancreatic cancer (Fig. 4A). The TNT index was assessed at 24, 48, 72, and 96 hours after initial incubation (median values are shown in Fig. 4B,C; raw data are provided in Supplementary Table 1). At 24 hours, none of the comparisons of TNT index was statistically significant. However, we did observe a heterogeneous response to variable concentrations of this drug at 48 and 72 hours. P-values from direct comparisons of S2013 TNT index data at 48 hours are presented in Supplementary Table 2. We detected a peak in TNT index occurring at the 800 ng/ml dose by 72 hours for both cell lines (Fig. 4B,C). The difference in TNT formation between the most stimulating (800 ng/ml) as compared to that for the least stimulating (1200 ng/ml) doses was 3-fold by this last time-point in the S2013 cell line (p = 0.02) and 1.5-fold in the Mia PaCa-2 cell line (p < 0.0001). There was also a significant difference noted in the S2013 TNT index between 800 ng/ml and 1000 ng/ml at 72 hours (p < 0.0001).

For MIA PaCa-2, significant differences in TNT index were observed when comparing the following doses at the 24-hour timepoint: 0 and 1200 ng/ml (p = 0.002); 200 and 600 ng/ml (p < 0.0001); 400 and 600 ng/ml (p = 0.01); 400 and 1200 ng/ml (p = 0.03); 600 and 800 ng/ml (p = 0.01); 600 and 1000 ng/ml (p < 0.0001); 600 and 1200 ng/ml (p < 0.0001); and 800 and 1200 ng/ml (p = 0.0001). P-values for all comparisons by dose at 48 and 72 hours for this cell line can be found in Supplementary Table 3. Median cell proliferation values over time are provided in distribution box plot format in Supplementary Fig. 5, and direct statistical comparisons are provided in Supplementary Table 4.

### TNTs facilitate intercellular redistribution/efflux of doxorubicin between chemoresistant and chemosensitive cancer cells

TNTs are known to facilitate the intercellular transfer of many cytosolic components, including but not limited to mitochondria, Golgi vesicles, microRNAs, exosomes, and even nuclei^19,22,45–47^. Our group recently reported that TNTs can facilitate direct cell-to-cell transfer and redistribution of therapeutic cancer-targeting treatments specifically oncolytic viruses engineered to selectively target cancer cells^48^. Furthermore, we also demonstrated that after infection with an oncolytic virus, TNTs also mediated intercellular distribution of the viral thymidine kinase-activated nucleoside analog ganciclovir. This transport of cytotoxic drug from non-virus infected to virus-infected cells resulted in increased cell-kill^48^, representing a newly described form of bystander effect in the treatment of cancer.

**Figure 5:**
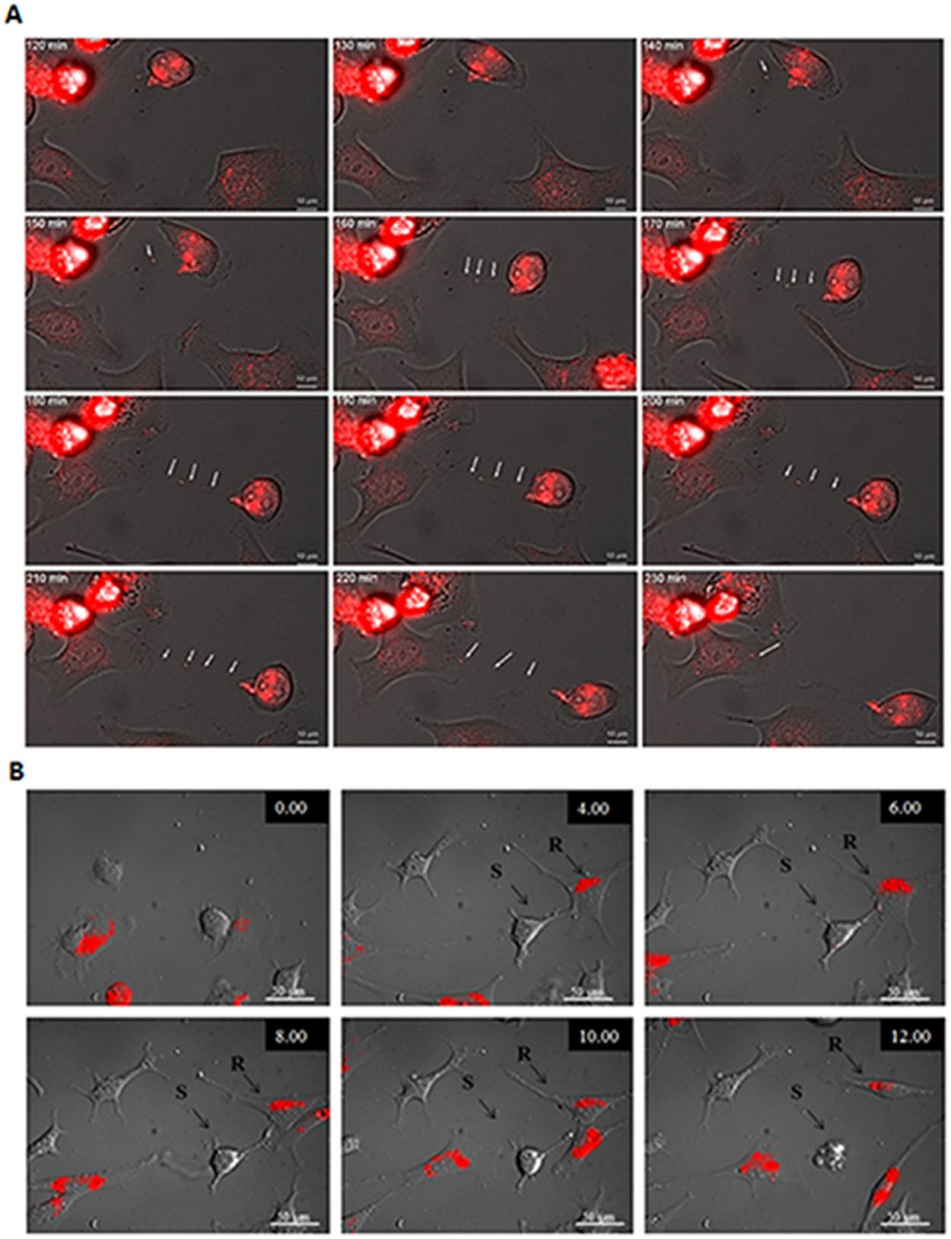
TNTs act as a direct conduit for intercellular transfer and redistribution of the chemotherapeutic drug doxorubicin. (**A**) TNT formation and intercellular transfer of doxorubicin between S2013 pancreatic adenocarcinoma cells. (**B**) Composite of serial images from time-lapse microscopy demonstrating intercellular transfer of doxorubicin from a chemoresistant ovarian cancer cell (SKOV3) to a chemosensitive cell (A2780) via a TNT, resulting in cell death of the chemosensitive cell. Images were taken every 15 minutes for 24 hours using a wide-field Zeiss Axio200M microscope.

If sensitive cells expel drugs to resistant cells via TNTs, this could potentially allow drug-treated cells to survive by minimizing drug exposure. Conversely, resistant cells expelling drugs to sensitive cells via TNTs could induce cell death, thereby enriching the population of drug-resistant cells and allowing them to thrive with reduced competition in the microenvironment. The chemotherapeutic drug doxorubicin, which was the focus of the current study, auto-fluoresces (excitation: 480 nm, emission: 560–590 nm) and can be easily examined using fluorescence microscopy^49^. Taking advantage of this drug’s unique property, we attempted to capture microscopy evidence of cancer cells harnessing TNTs as conduits for intercellular redistribution of doxorubicin after short-term treatment. We initially determined that S2013 cells readily internalized the doxorubicin via fluorescence microscopy (Fig. 5A). We then performed automated time-lapse microscopy by taking images every 10 minutes for 5 hours to track movement of the drug. We confirmed the formation of TNTs and the successful transfer of doxorubicin from doxorubicin-positive cells to TNT-connected recipient cells (composite images and schematic shown in Fig. 5A and Supplementary Movie 1). To quantify the relative amount of transfer of this auto-fluorescing drug, we analyzed the intensity of fluorescence within the TNT over time using ImageJ, reported as arbitrary units (a.u) (Supplementary Fig. 6). The transfer occurred between the time intervals of 160 and 250 minutes of the total duration of the time-lapse experiment. The total TNT fluorescence fluctuated over time, providing further supportive evidence that the drug was being transported rather than remaining stationary within the TNT.

To confirm this finding in an additional cancer model, we repeated the experiment using chemoresistant ovarian cancer cells that form TNTs at differential rates based on chemoresistant vs chemosensitive status of co-cultured cells^32^. In this experiment, we treated SKOV3 (multi-drug resistant ovarian carcinoma cells) with doxorubicin and co-cultured them with untreated A2780 cells, which are known to be sensitive to doxorubicin. Using time-lapse microscopy, we analyzed images for TNT formation between the cell populations. In one particularly striking instance, we observed transfer of fluorescing doxorubicin granules from a SKOV3 cell to an A2780 cell via a TNT (Fig. 5B, and Supplementary Movie 2). Notably, only a minimal amount of effluxed drug was required for the recipient chemosensitive A2780 cell to undergo cell death within 3 hours of drug transfer via the TNT.

## Discussion

Chemoresistance remains a significant clinical problem, yet the underlying cellular mechanisms remain unclear despite advances in the field of cancer treatment. Our studies were designed to evaluate the formation of TNTs — a unique form of cellular protrusion implicated in long-distance cell communication — as a cellular stress response. We found that the response after exposure of pancreatic cancer cells to doxorubicin was dose-dependent, and that TNTs facilitate a novel and direct form of cell-to-cell drug efflux among pancreatic and ovarian cancer cells through transport of a cytotoxic drug (doxorubicin). To our knowledge, direct cell-to-cell transfer and efflux of chemotherapy agents via TNTs has been reported in one other instance, in an *in vitro* model of acute myelogenous leukemia. In that study, researchers detected localization of the drug daunorubicin to lysosomes and visualized cell-to-cell transfer of the drug via TNTs^50^. In 2012, we reported that TNTs, or at least TNT-like structures, could be visualized connecting cells in intact tumors resected from human patients with malignant pleural mesothelioma^19,31^. Additional tumor types for which we have reported similar findings include lung carcinomas, ovarian carcinomas, osteosarcomas, breast carcinomas, neuroendocrine tumors, and colon cancers^19,32,47,51^. Here, we provide visual evidence that pancreatic adenocarcinomas as well as neuroendocrine carcinomas can be added to this growing list of aggressive cancer types that are capable of forming TNTs for mediating long-range intercellular communication *in vivo*. Detection of TNTs, or TNT-like structures, in this setting further supports the notion that the *in vitro* findings reported in this paper have potential clinical relevance.

We used doxorubicin, an anthracycline chemotherapeutic drug in extensive clinical use for a variety of epithelial malignancies including breast and ovarian cancers, because of its wide use in cancer chemotherapy-based treatment and *in vitro* autofluorescent properties^49^. Its primary mechanism of action involves DNA intercalation and topoisomerase inhibition. Our observations underscore a possible role for TNTs in drug-efflux of either chemo-naïve or in more advanced chemorefractory cancers. We exposed cells to varied concentrations of this drug (200, 400, 600, 800, 1000, and 1200 ng/mL) and compared the results to no drug. In a clinical setting, doxorubicin follows a fast distribution phase followed by a slow elimination phase; the drug reaches a peak concentration of ~600 ng/ml before being distributed to other tissues after intravenous administration^52,53^. Adding an optimized concentration of the drug (low enough not to induce immediate toxicity leading to cell death, but high enough to be detected using fluorescent microscopy) allowed us to perform time-lapse imaging over 24–48 hours. We visualized direct outgrowth of TNTs from drug-treated chemoresistant (SKOV3) cells to chemosensitive (A200) ovarian cancer cells, confirming that resistant cells were capable of initiating this unique form of cellular interaction. After co-culture of doxorubicin-treated SKOV3 cells with A2780 cells, we visualized a TNT connecting these cells and facilitating transfer of a minimal amount of this autofluorescent drug. Within hours, the recipient chemosensitive (A2780) cell involuted and underwent cell death. As we have previously reported, when these cells were co-cultured, formation of TNTs from chemoresistant to chemosensitive ovarian cancer cells constituted the fewest number of interactions, as compared with sensitive-to-resistant, resistant-to-resistant, or sensitive-to-sensitive^32^. The finding that a drug can be redistributed via TNTs poses a potential new paradigm for cellular mechanisms of drug efflux and the development of drug resistance in cancers. We speculate that this finding suggests TNTs may serve as an alternative mechanism capable of exporting chemotherapeutic drugs in an efficient manner between connected cells.

In 2013, we published a review of the potential impact of intercellular communication on tumor heterogeneity in pancreatic cancer^54^. At that time, we speculated on the role that TNTs could play in tumor-tumor interactions in this particularly heterogeneous tumor microenvironment. Our current study supports the notion that TNTs represent a non-genetic intercellular stress response by which tumors can survive despite exposure to cytotoxic chemotherapeutic drugs in a hypoxic tumor microenvironment. In 2015, Ware *et al*. reported a similar finding of upregulated TNT formation between malignant pancreatic cells following a different therapeutic modality (radiofrequency ablation; RFA)^55^. In examining the percent of TNT-forming cells prior to and following RFA, the investigators discovered significantly increased formation of TNTs in PANC-1 and AsPc-1 malignant cells and, as in our case, proposed this action as a stress response following that form of treatment. Interestingly, they too used HPDE as a non-malignant comparison, and the percent of TNT-forming cells was relatively negligible both before and after RF treatment^55^. The findings from both studies support the notion that physiologic or metabolic stressors in the microenvironment can induce or activate other forms of cellular protrusions as well, including invadopodia responsible for invasive cancer cell migration^56^. Based on these as well as our findings, we speculate that the ability of TNTs to form in response to stress induced by either chemotherapeutic drug or other non-pharmacologic interventions may, at least in part, explain the early emergence of drug resistance. Further, the ability of TNTs to mediate intercellular efflux, or redistribution, of drugs — potentially to subtherapeutic levels — may allow cells connected via these cellular ‘networks’ to protect each other from drug susceptibility. In this potential model, with continued administration of chemotherapy over time, the cells that adapt best to chemotherapeutic stress would survive and overtake other clonal subpopulations.

There is precedence for evolution of drug efflux in cancer cells, as development of chemoresistance via overexpression of P-glycoprotein (P-gp) is already well established^57^. This protein is a member of the ABC transporter family; when overexpressed, it becomes embedded within the plasma membrane, serving effectively as an ATP-dependent pump for efflux of chemotherapeutic agents^58,59^. The potential for intercellular transfer of P-gp to occur, and for the protein to remain functional after this horizontal transfer, has also been demonstrated^18,60^. Furthermore, P-gp has been studied in the context of TNTs, and specifically in ovarian cancer^61^. TNT-mediated transfer of both mitochondria and P-gp has been demonstrated in breast and ovarian cancer cell lines, in addition to transfer mediated without cell-to-cell contact via microparticles; this transfer has been associated with chemoresistance^18,45,61^. Several recent studies have in fact confirmed cell-to-cell transport of chemotherapeutic drugs via cellular microparticles or exosomes^62^. As compared with TNTs, exosomes are diffusible vessels of cell transport but may not be as efficient for cargo delivery as TNTs, which act as nanoscale pipelines between distant cells that cannot be connected via connexin-lined (gap junction) channels. Our results support the notion that TNTs represent a newly identified mechanism of drug efflux, which comprises both cytoplasm-to-cytoplasm extrusion of drug via TNTs. Intercellular transfer of P-gp has been examined in other cancer models (e.g., in bladder cancer); at least one study concluded that cell-to-cell contact is not necessary for this transfer to take place and mediate drug resistance^63^. The authors concluded microparticles were the most likely mode of transfer in that study. In this context, TNT-mediated transfer of P-gp may play a more complementary or secondary role to cytoplasm-to-extracellular environment extrusion via P-gp/multidrug resistance (MDR) channels.

We have shown that TNT formation is significantly higher in malignant pancreatic cells as compared to pancreatic ductal (non-malignant) epithelial cells. This result and difference in TNTs between malignant and benign cells is consistent with our data in other invasive cancers, such as malignant pleural mesothelioma among others^31^. We have previously speculated that TNTs play a role in cancers that may allow us to harness them as conduits for more effective drug delivery^51,61^. This concept would be particularly intriguing and applicable to pancreatic cancer, in which effective drug penetration remains a challenge due to dense desmoplastic reaction and thick hyaluronan coats that preclude diffusion of drugs into tumors^54,62^. Here, we found that the length of TNTs was notably much longer in pancreatic cancer cell lines cultured *in vitro*, as compared to their counterparts within human tumors. We speculate that this finding is due to the magnitude of this stromal density *in vivo*.

Interestingly, TNTs have been shown to provide mesenchymal stem cell rescue of cardiomyocytes damaged after exposure to anthracycline drugs by TNT-mediated transfer of mitochondria^64^. In the setting of cancer, the effects may actually be reversed, in which chemotherapeutic damage and the resultant rise in TNT formation leads to redistribution of the drug to other cells — in an effort for the affected cells to save themselves — or induce a stress response that leads damaged cells to “offer” their mitochondria and other vital cell components to other neighboring cells as a “sacrifice” to benefit the overall cancer cell population. The niche of TNTs and drug delivery is thought-provoking and one that we predict will gain increased interest.

In summary, TNT formation as a response to chemotherapeutic drug exposure is dose-dependent, with the highest rate of formation occurring at physiologically relevant concentrations. This differential response represents a potential cellular stress response that facilitates increased rates of intercellular TNT-mediated communication and transfer of cytotoxic drugs, such as doxorubicin. We postulate that this novel form of long-range drug efflux redistributes these drugs, and may or may not result in cell death of chemosensitive recipient cells. This study provides preliminary insight into the potential pathophysiologic role of TNTs in facilitating drug redistribution, and their formation as a stress response, and will need to be substantiated through further in-depth investigations. In this context, TNTs may provide a potential new and additional mechanism by which cells propagate drug chemoresistance in invasive cancers.

## Materials and Methods

### Cell lines and cell culture

MIA PaCa-2 (ATCC CRL-1420)^65^, S2013 (also known as S2-013 or SUIT.2013^66,67^, CVCL_B280), CAPAN-1 (ATCC HTB79)^65^, and CAPAN-2 (ATCC HTB-80)^65^ cells were derived from human pancreatic adenocarcinoma, and HPDE cells from human ductal epithelium (CVCL_4376)^63^. MIA PaCa-2 and CAPAN-2 are derived from primary pancreatic tumors; S2013 and CAPAN-1 are derived from metastatic tumors. S2013 was obtained from ThermoFisher Scientific (Grand Island, NY); MIA PaCa-2, CAPAN-1, and CAPAN-2 cell lines were obtained from the American Type Culture Collection (ATCC).

A2780 and SKOV3 cell lines were kindly provided by Dr. Sundaram Ramakrishnan at the University of Minnesota. A2780 is a malignant human epithelial cell line derived from an endometrioid ovarian tumor. SKOV3 cells were derived from the ovarian adenocarcinoma ascites (peritoneal metastasis) of an untreated patient. SKOV3 cells are resistant to cisplatin, TNF, diphtheria toxin, and doxorubicin^24^. Cell lines were authenticated using sequence tandem repeat genotype profiling (Johns Hopkins University, STR Profiling for Human Cell Line Authentication) (MIA PaCa-2, S2013) or used immediately following acquisition from ATCC (CAPAN-2). Ovarian cancer cell genotypes were confirmed by comparison to available genetic profiles using the University of Colorado database (website: http://dnasequencingcore.ucdenver.edu/pdf-Files/Korch%20et%20al%20-%20Table%20S4%20Ovarian%20profiles.pdf).

All cancer cell lines were cultured in RPMI-1640 medium (Thermo Fisher Scientific, Waltham, MA, USA). All media was supplemented with 10% (v/v) fetal bovine serum (FBS) and 1% (v/v) Antibiotic-Antimycotic (Thermo Fisher Scientific, Waltham, MA, USA) at 37 °C in a humidified 5% CO2/95% air atmosphere. HPDE cells were cultured in defined keratinocyte medium supplemented by epidermal growth factor and bovine pituitary extract (Invitrogen Catalog #: 17005042) (Life Technologies, Inc., Grand Island, NY), with 1x antibiotic-antimycotic (Gibco Catalog #15240-062); for Supplementary Fig. 4, this medium is referred to as the “native culture medium.”

Cell lines were passaged every 2–3 days using trypsin-0.53 mM EDTA solution, kept in T-75 cm^2^ tissue culture flasks, and confirmed to be negative for mycoplasma infection. To stimulate TNT formation for *in vitro* examination, cells were grown in what our group refers to as “TNT-inducing medium”^19^ [2.5% FCS in RPMI-1640 containing 50 mM glucose, supplemented with 1% P-S, 2% L-glutamine with 10 mM ammonium lactate] (Sigma Aldrich, St. Louis, Missouri) and acidification of medium to pH 6.6, per our prior study^19^. Cell cultures were done in 75 cm^2^ tissue culture flasks (Falcon, Becton Dickson, Oxnard, CA) at 37 °C in 5% CO_2_.

### Quantification of TNT index

TNTs were visually identified and quantified as previously described^19,25,31,46^. Briefly, these parameters included (i) lack of adherence to the substratum of tissue culture plates, including visualization of TNTs passing over adherent cells; (ii) TNTs connecting two cells or extending from one cell were counted if the width of the extension was estimated to be <1000 nm; and (iii) a narrow base at the site of extrusion from the plasma membrane. Cellular extensions not clearly consistent with the above parameters were excluded. An Olympus IX70 inverted microscope (Olympus Corporation) with 20x objective lens was used to count the number of TNTs and cells in 10 randomly chosen fields of each 6-well plate at 24, 48, 72, and 96 hours. Representative images of each field were taken at all time points in each well. Experiments were performed in triplicate for each cell line. To determine TNT formation, cells were plated at a density of 2.5 × 10^5^ cells/well in 6-well adherent tissue culture plates (Fisher Scientific, Pittsburgh, PA) at 37 °C in 5% CO_2_ with TNT-inducing medium^19^. TNTs and cells were counted manually, and the TNT index was calculated as the number of TNTs per cell (TNTs/cell) using previously described methods^31^.

### Pharmacologic treatment of cell lines

Doxorubicin (Doxo) (D-4000, LC Laboratories) is an anthracycline chemotherapeutic drug. It was used in this study for its autofluorescent properties.

### Incubation with doxorubicin

MIA PaCa-2 and S2013 cells were cultured in T-flasks before trypsinization. Cells were then treated for 8 minutes in suspension with either 0, 200, 400, 600, 800, 1000, or 1200 ng/mL doxorubicin (D-4000, LC Laboratories, Woburn, MA) in RPMI media. Cells were then washed, pelleted, and plated into 12-well plates (0.1 × 10^6^ cells/well), marking time point 0 hours. Cells were cultured at 37 °C in a humidified 5% CO_2_/95% air atmosphere for either 24, 48, or 72 hours. A2780 and SKOV3 cell lines were treated with doxorubicin for time-lapse imaging experiments. Doxorubicin was used at optimized concentrations to maximize its autofluorescent properties.

### Imaging and TNT quantification

An Olympus IX70 inverted microscope (Olympus Corporation, Waltham, MA) with 20x objective lens was used to identify TNTs using the aforementioned criteria via live imaging. For each cell line, concentration, and time point, 3 fields of view (FOV) were taken from each of 5 wells, in triplicate.

### Time-lapse microscopy

To visualize doxorubicin transfer via TNTs, we performed time-lapse microscopy imaging every 15 minutes for 24 hours (ovarian cancer experiment) or every 10 minutes for 5 hours (pancreatic cancer experiment). Cells were first allowed 12 hours to settle and adhere to the plates prior to imaging. For time-lapsed imaging, multiple 20x fields of view containing evenly distributed cells were chosen using a wide-field Zeiss Axio200M microscope costume-fitted with a stage incubator that maintains environmental conditions at 37 °C and 5% CO_2_.

### Quantification of doxorubicin intensity

Individual images were selected from the time-lapse experiment that documented intercellular of transfer of doxorubicin between pancreatic cancer cells via a TNT. The images were selected based on the times that doxorubicin could be visually identified within the TNT (between timepoints 160 and 250 minutes) and analyzed using ImageJ software. The TNT was traced and marked using the Freehand Selection tool of ImageJ, which measured the total TNT fluorescence based on examination of pixels, reported as an arbitrary unit which we graphed as shown in Supplementary Fig. 6.

### Imaging TNTs/TNT-like structures in pancreatic tumor specimens

#### Ethical approval and informed consent

Tumor specimens from four patients with pancreatic cancer were obtained from the University of Minnesota (UMN) Tissue Procurement Facility under the auspices of a UMN IRB-approved protocol. Informed written consent was obtained, and patient identifiers were removed to ensure anonymity. All procedures for tumor procurement were in compliance with ethical regulations for obtaining and using human tissue.

The images shown in Fig. 1 represent our initial attempt to image TNTs in pancreatic tumor tissue from a human patient. Tumor sections (100–300 mm thick) were cut using a Vibratome and then stained using Hoechst 33342 (10 mg/ml) and MitoTracker Orange dyes (500 nM). Stained sections were mounted between two glass coverslips and imaged as previously described. 3D reconstructions were performed using Nikon NIS elements AR software.

We further refined the protocol to improve detection of TNTs in context of the dense and highly stromatous tumor microenvironment. For the images shown in Fig. 2, formalin-fixed pancreatic tumor specimens from three additional patients were submerged in optimal cutting temperature (OCT) embedding medium and placed on dry ice for three minutes. Specimen-OCT blocks were cut into 100 µm and 300 µm sections with a Leica CM1850 cryostat. Sections were added to 9-well PYREX™ glass plates and rinsed three times with 1X Phosphate-Buffered Saline (PBS). Our lab investigated how to optimize a new tumor imaging protocol, so staining solution reagent concentrations and cryostat slice thickness varied. Tumors were carefully sectioned to either 100 µm or 300um slices as indicated in the corresponding figure legend, and stained using either Alexa Fluor™ 488 Phalloidin or MitoTracker™ Orange CMTMRos per manufacturer’s instructions. All staining solutions contained 1% donkey serum (D9663 Sigma-Aldrich), 0.1% Triton-X in PBS, and 0.12 µM PureBlu™ Hoechst 33342 dye. After applying staining solutions, PYREX plates were covered with aluminum foil for ten minutes. To make agar, 0.38 g noble agar and 25 mL distilled H_2_O were added to an Erlenmeyer flask and placed in a beaker of boiling water until dissolved. Tumor sections were mounted to glass coverslips and placed in a coverslip boat rack. To dehydrate, coverslip boats were immersed in 75% EtOH within a glass container for 30 minutes. This step was repeated twice more with 95% and 100% EtOH. DPX mounting medium was generously applied to pre-labeled microscope slides. Tissue-mounted coverslips were then mounted to slides and allowed to dry undisturbed, protected from light for 48 hours. Slides were imaged with the Nikon A1R Multiphoton confocal microscope at the University of Minnesota Imaging Center. Parameters for 3-dimensional imaging included obtaining 40 z-stack slices, with 0.3 µm step size.

### Statistical Analysis

TNT indices and lengths were not normally distributed and therefore Wilcoxon Rank Sum tests were used to compare TNT indices and lengths for each combination of dose and time measurements within each cell line. P-values were conservatively adjusted for multiple comparisons within each experiment using Bonferroni correction. Analyses were conducted in SAS version 9.4 (Cary, NC), and *p*-values < 0.05 were considered statistically significant.

### Data availability

Supporting data are available in the main text and in the Supplementary Online Files section, and upon request.

### Ethical approval and informed consent

As stated in further detail in Material and Methods: the use of human tumor samples was carried out in accordance with relevant guidelines and regulations, with approval from the University of Minnesota’s Institutional Review Board (IRB). Informed consent was obtained from all participants.

## Electronic supplementary material

Supplementary Information

Supplementary Movie S1

Supplementary Movie S2
