# Supplementary material for: Chemotherapy-Induced Tunneling Nanotubes Mediate Intercellular Drug Efflux in Pancreatic Cancer
Source: Sci Rep. 2018 Jun 21;8:9484. doi: 10.1038/s41598-018-27649-x (PMC6013499; doi:10.1038/s41598-018-27649-x)
Supplement: Supplementary file 1 — Supplementary Information [file 41598_2018_27649_MOESM1_ESM.pdf]

SUPPLEMENTARY INFORMATION

Chemotherapy-Induced Tunneling Nanotubes Mediate Intercellular Drug Efflux in Pancreatic Cancer

Snider Desir[1,2*], Patrick O'Hare[1*], Rachel Isaksson Vogel[3], William Sperduto[1], Akshat Sarkari[1], Elizabeth L Dickson[3], Phillip Wong[1,4], Andrew C Nelson[5], Yuman Fong[6], Clifford J Steer[4], Subbaya Subramanian[7], Emil Lou[1,2]

[1]Department of Medicine, Division of Hematology, Oncology and Transplantation, University of Minnesota, Minneapolis MN 55455
[2]Department of Integrative Biology and Physiology, University of Minnesota, Minneapolis MN 55455
[3]Department of Obstetrics and Gynecology, Division of Gynecologic Oncology, University of Minnesota, Minneapolis, MN 55455
[4]Department of Medicine, Division of Gastroenterology, Hepatology and Nutrition, University of Minnesota, Minneapolis MN 55455
[5]Department of Laboratory Medicine & Pathology, University of Minnesota, Minneapolis MN 55455
[6]Department of Surgery, City of Hope Medical Center, Duarte CA 91010
[7]Department of Surgery, University of Minnesota, Minneapolis MN 55455

*These authors contributed equally to this work

Corresponding author:
Emil Lou, M.D., Ph.D
Division of Hematology, Oncology and Transplantation
Mayo Mail Code 480
420 Delaware Street SE
Minneapolis, MN 55455
E-mail: emil-lou@umn.edu / Phone: (612) 624-5944 / Fax: (612) 625-6919

SUPPLEMENTARY FIGURE LEGENDS

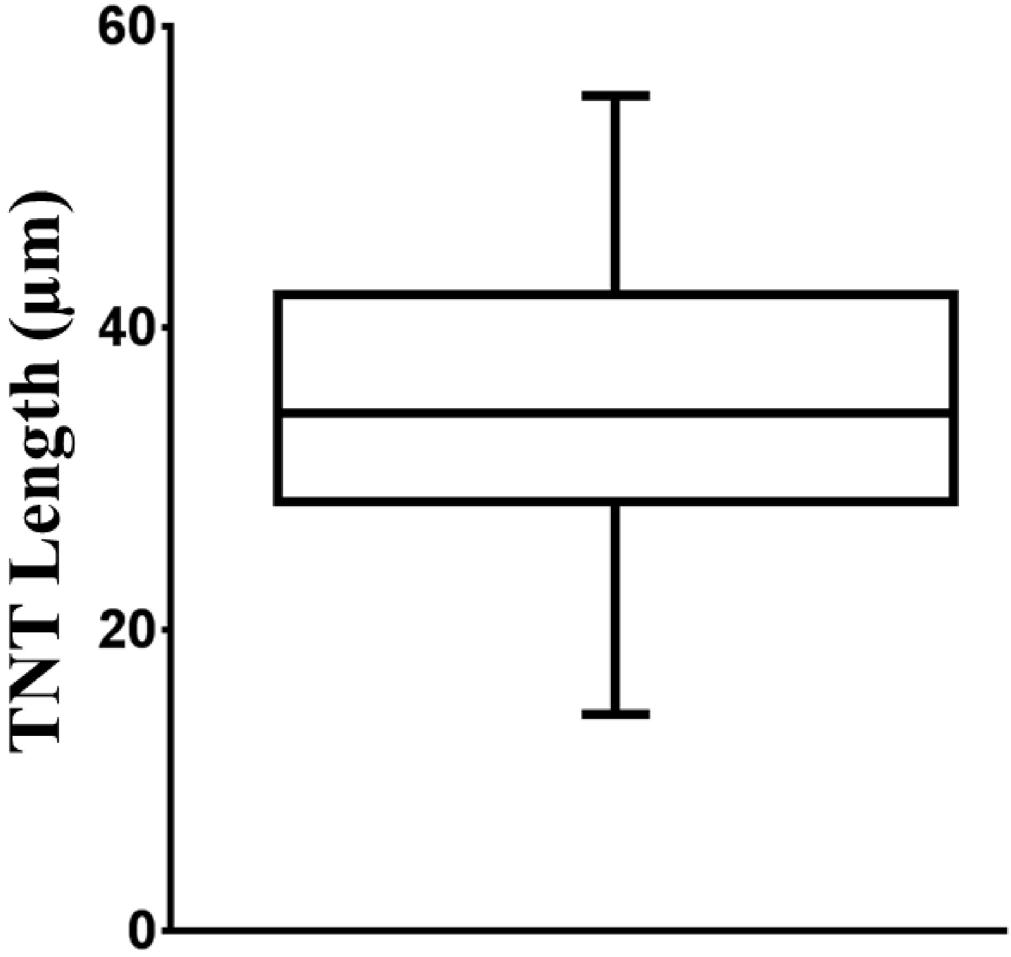

Supplementary Figure 1. Box plot distribution of lengths of TNTs imaged in resected pancreatic tumors. TNT length measurements were taken from pancreatic tumor images in three different patients (n = 14 images). All images were acquired at a 25x objective with water immersion. Images were analyzed using FIJI software using a known scale bar length of five microns (5 µm). Using a conversion factor between the number of microns on the scale bar and its corresponding pixel length, TNT length in every image was correspondingly measured. The line tool in FIJI was further used to measure the lengths of TNTs in each image. TNT lengths are presented using a box

and whisker plot. The line in the middle of the box plot represents the median, edges of the box correspond to the first and third quartiles, and the whiskers represent the minimum and maximum.

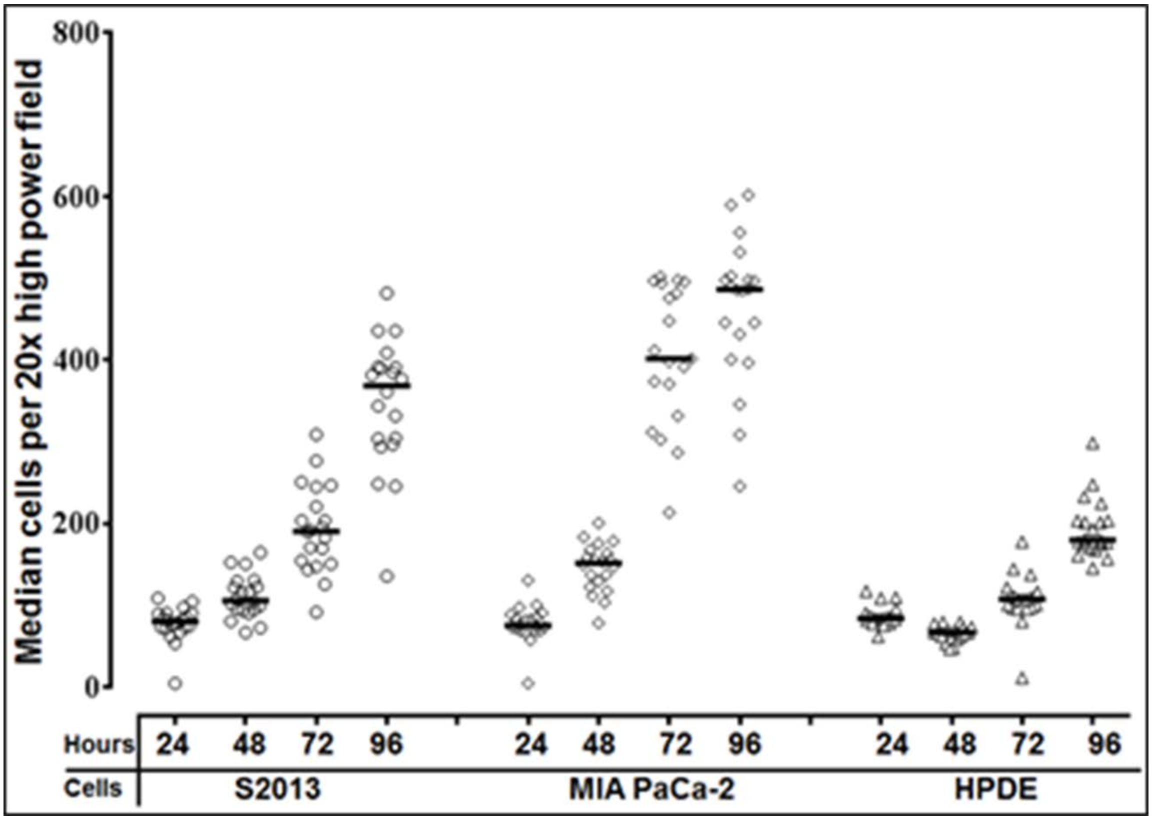

Supplementary Figure 2. Number of cells over time for the S2013, MIA PaCa-2, and HPDE cell lines at 24, 48, 72 and 96 hours. The symbols represent individual data points, and the solid lines represent the median.

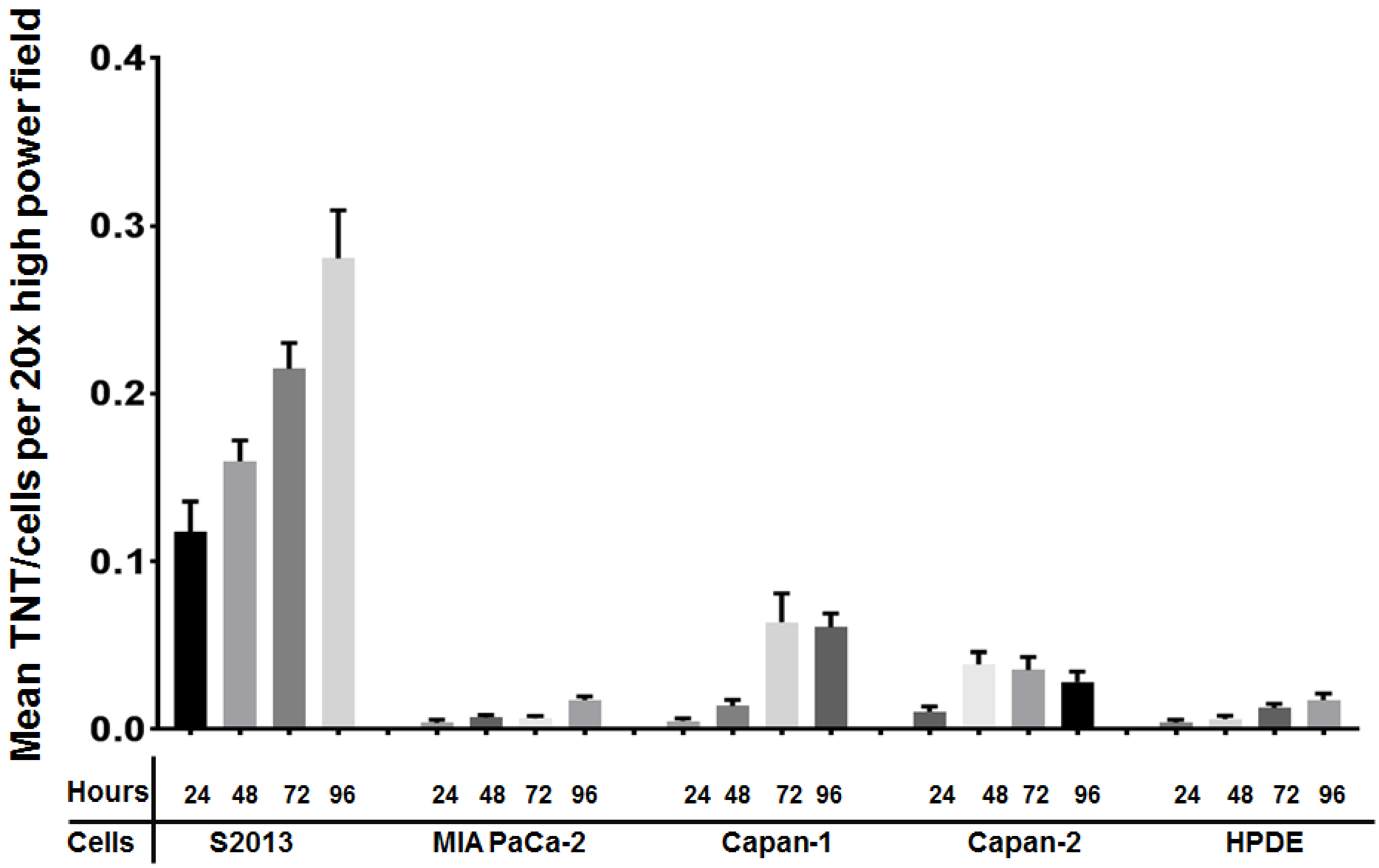

Supplementary Figure 3. TNT formation amongst pancreatic cancer cells and HPDE cells, depicted here as the mean number of TNTs/cell. Note that these data are depicted as median values in the box plots shown in Figure 3 in the main text.

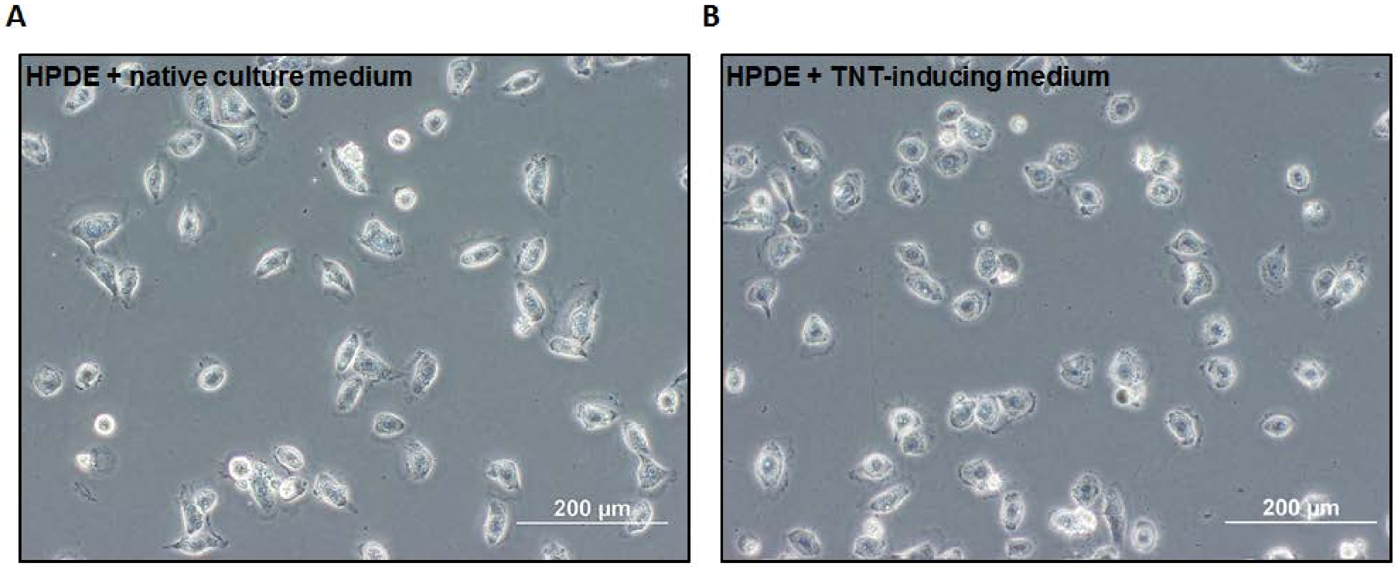

Supplementary Figure 4. Representative images of TNTs connecting human pancreatic ductal epithelial (HPDE) cells cultured in (A) native culture medium [Keratinocyte SFM, + EGF + bovine pituitary extract with 1x antibiotic-antimycotic] compared with the same cell type cultured in (B) TNT-inducing medium [2.5% FCS in RPMI-1640 containing 50 mM glucose, with 1% penicillin-streptomycin, 2% L-glutamine with 10 mM ammonium lactate and acidification of medium to pH 6.6].

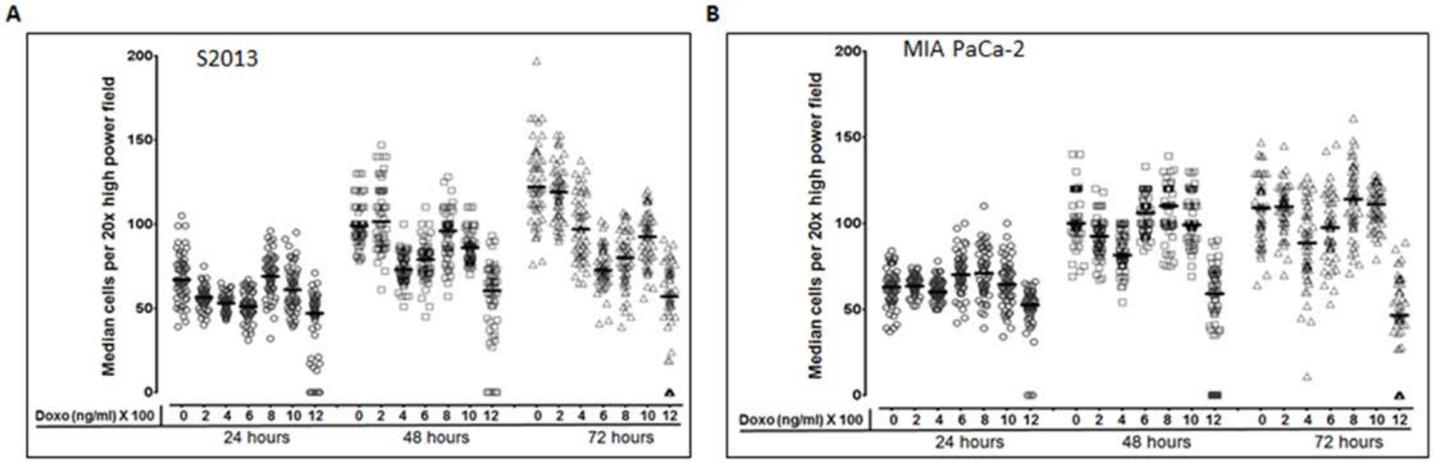

Supplementary Figure 5. Number of cells over time after exposure to six concentrations of doxorubicin for the S2013 (A) and MIA PaCa-2 (B) cell lines at 24, 48, 72 and 96 hours. The symbols represent individual data points, and the solid lines represent the median.

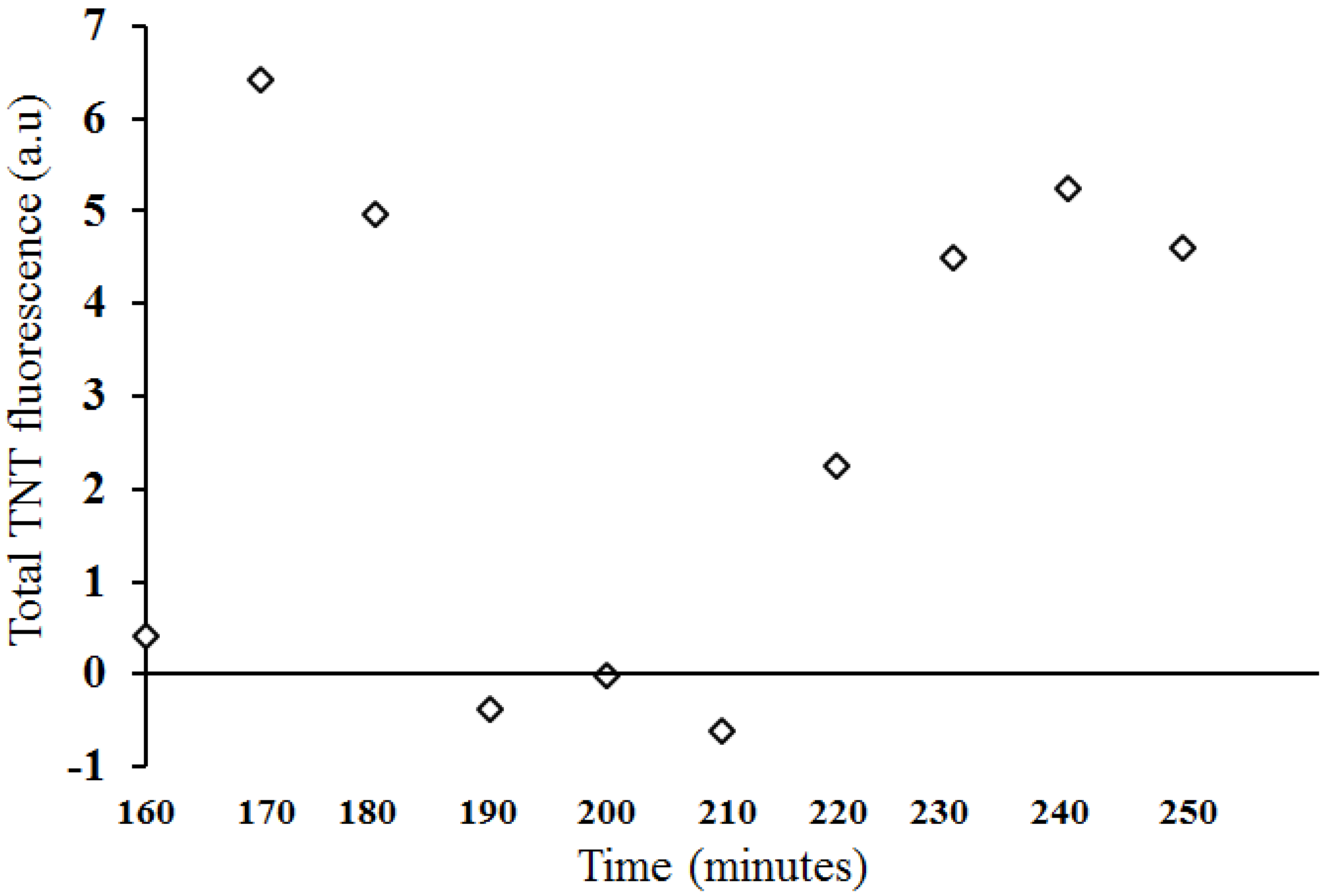

Supplementary Figure 6. Quantification of intensity of autofluorescing doxorubicin over time during intercellular transfer of this drug within a TNT connecting S2013 pancreatic carcinoma cells. The intensity was assessed and reported as arbitrary units (a.u.) using ImageJ software.

SUPPLEMENTARY MOVIE LEGENDS

Supplementary Movie S1: Video of time-lapse microscopy demonstrating TNT formation and intercellular transfer of doxorubicin between S2013 pancreatic adenocarcinoma cells.

Supplementary Movie S2: Video of time-lapse microscopy demonstrating intercellular transfer of doxorubicin from a chemoresistant ovarian cancer cell (SKOV3) to a chemosensitive cell (A2780) via a TNT, resulting in cell death of the chemosensitive cell. We observed one such instance in which drug efflux took place. This suggests that while TNT-mediated drug efflux may not be a common event, it does occur and has implications for how we should comprehensively investigate intra- and extracellular redistribution of chemotherapeutic drugs. This observation provides an interesting, preliminary observation of direct cell-to-cell efflux of a cytotoxic chemotherapeutic drug via TNTs.

TABLE AND LEGENDS

Supplementary Table 1. Median number of TNTs/cell for S2013 and MIA PaCa-2 cell lines exposed to various concentrations of doxorubicin.

**S2013**			
Doxo (ng/ml)	24 hours	48 hours	72 hours
0	0.026	0.025	0.045
200	0.018	0.050	0.072
400	0.020	0.024	0.068
600	0.038	0.041	0.078
800	0.024	0.061	0.093
1000	0.016	0.061	0.046
1200	0.000	0.034	0.025

**MIA PaCa-2**			
Doxo (ng/ml)	24 hours	48 hours	72 hours
0	0.022	0.040	0.058
200	0.016	0.051	0.054
400	0.017	0.049	0.057
600	0.025	0.053	0.061
800	0.018	0.066	0.073
1000	0.015	0.077	0.051
1200	0.020	0.054	0.056

Supplementary Table 2. P-values for comparisons of S2013 TNT indices by doxorubicin dose at 48 hours (Figure 4B). P-values adjusted for multiple comparisons using a Bonferroni correction**.**

**Dose**	2	4	6	8	10	12
0	0.03	0.02	<0.0001	<0.0001	<0.0001	0.03
2		1.00	1.00	0.004	<0.0001	1.00
4			0.98	<0.0001	<0.0001	1.00
6				0.07	<0.0001	1.00
8					1.00	0.34
10						0.004

Supplementary Table 3. P-values for comparisons of MIA PaCa-2 TNT indices by doxorubicin dose at 48 and 72 hours (Figure 4C). P-values adjusted for multiple comparisons using a Bonferroni correction**.**

**Dose**	2	4	6	8	10	12
0	<0.0001	1.00	<0.0001	<0.0001	<0.0001	0.03
2		<0.0001	0.36	1.00	1.00	0.03
4			<0.0001	<0.0001	<0.0001	<0.0001
6				<0.0001	<0.0001	1.00
8					1.00	<0.0001
10						<0.0001

**Dose**	2	4	6	8	10	12
0	<0.0001	<0.0001	<0.0001	<0.0001	1.00	<0.0001
2		1.00	1.00	0.06	<0.0001	<0.0001
4			1.00	0.002	<0.0001	<0.0001
6				1.00	<0.0001	<0.0001
8					<0.0001	<0.0001
10						<0.0001

Supplementary Table 4. P-values for comparisons of cell proliferation by doxorubicin dose at 24, 48 and 72 hours for S2013 and MIA PaCa-2 (Supplementary Figure 5). P-values adjusted for multiple comparisons using a Bonferroni correction.

A. S2013

24 hours – adjusted p-values

**Dose**	2	4	6	8	10	12
0	<0.0001	<0.0001	<0.0001	1.00	0.52	<0.0001
2		0.02	0.02	<0.0001	0.96	<0.0001
4			1.00	<0.0001	<0.0001	0.04
6				<0.0001	<0.0001	0.40
8					0.12	<0.0001
10						<0.0001

48 hours – adjusted p-values

**Dose**	2	4	6	8	10	12
0	1.00	<0.0001	<0.0001	1.00	<0.0001	<0.0001
2		<0.0001	<0.0001	0.16	<0.0001	<0.0001
4			0.95	<0.0001	<0.0001	<0.0001
6				<0.0001	0.004	<0.0001
8					0.11	<0.0001
10						<0.0001

72 hours – adjusted p-values

**Dose**	2	4	6	8	10	12
0	1.00	<0.0001	<0.0001	<0.0001	<0.0001	<0.0001
2		<0.0001	<0.0001	<0.0001	<0.0001	<0.0001
4			<0.0001	<0.0001	1.00	<0.0001
6				1.00	<0.0001	<0.0001
8					0.006	<0.0001
10						<0.0001

B. MIA PaCa-2

24 hours – adjusted p-values

**Dose**	2	4	6	8	10	12
0	1.00	1.00	0.04	0.08	1.00	<0.0001
2		0.25	0.004	0.05	1.00	<0.0001
4			<0.0001	<0.0001	1.00	<0.0001
6				1.00	0.56	<0.0001
8					0.64	<0.0001
10						<0.0001

48 hours – adjusted p-values

**Dose**	2	4	6	8	10	12
0	<0.0001	<0.0001	1.00	1.00	1.00	<0.0001
2		0.004	<0.0001	<0.0001	0.01	<0.0001
4			<0.0001	<0.0001	<0.0001	<0.0001
6				1.00	1.00	<0.0001
8					1.00	<0.0001
10						<0.0001

72 hours – adjusted p-values

**Dose**	2	4	6	8	10	12
0	1.00	<0.0001	0.11	1.00	1.00	<0.0001
2		<0.0001	0.01	1.00	1.00	<0.0001
4			1.00	<0.0001	<0.0001	<0.0001
6				<0.0001	<0.0001	<0.0001
8					1.00	<0.0001
10						<0.0001
